# Massively-parallel sequencing of genes on a single chromosome: a comparison of solution hybrid selection and flow sorting

**DOI:** 10.1186/1471-2164-14-253

**Published:** 2013-04-15

**Authors:** Jamie K Teer, Jennifer J Johnston, Sarah L Anzick, Marbin Pineda, Gary Stone, Paul S Meltzer, James C Mullikin, Leslie G Biesecker

**Affiliations:** 1 National Human Genome Research Institute, National Institutes of Health, Bethesda, MD, USA; 2 National Cancer Institute, National Institutes of Health, Bethesda, MD, USA; 3 Current address: H. Lee Moffitt Cancer Center and Research Institute, Tampa, FL, USA

**Keywords:** Flow sorting, Flow cytometry, Targeted-sequencing, Sequencing, Genomic-capture, Chromosome, Genome

## Abstract

**Background:**

Targeted capture, combined with massively-parallel sequencing, is a powerful technique that allows investigation of specific portions of the genome for less cost than whole genome sequencing. Several methods have been developed, and improvements have resulted in commercial products targeting the human or mouse exonic regions (the exome). In some cases it is desirable to custom-target other regions of the genome, either to reduce the amount of sequence that is targeted or to capture regions that are not targeted by commercial kits. It is important to understand the advantages, limitations, and complexity of a given capture method before embarking on a targeted sequencing experiment.

**Results:**

We compared two custom targeted capture methods suitable for single chromosome analysis: Solution Hybrid Selection (SHS) and Flow Sorting (FS) of single chromosomes. Both methods can capture targeted material and result in high percentages of genotype identifications across these regions: 59-92% for SHS and 70-79% for FS. FS is amenable to current structural variation detection methods, and variants were detected. Structural variation was also assessed for SHS samples with paired end sequencing, resulting in variant identification.

**Conclusions:**

While both methods can effectively target genomic regions for genotype determination, several considerations make each method appropriate in different circumstances. SHS is well suited for experiments targeting smaller regions in a larger number of samples. FS is well suited when regions of interest cover large regions of a single chromosome. Although whole genome sequencing is becoming less expensive, the sequencing, data storage, and analysis costs make targeted sequencing using SHS or FS a compelling option.

## Background

The genome can be interrogated in a random (whole genome shotgun, WGS) or directed (targeted sequencing) manner. Each approach has advantages and disadvantages [1,2]. Targeted sequencing (or genomic capture) enriches a desired subset of a genome and therefore requires substantially less sequence to generate the needed coverage over the region of interest. As sequencing costs continue to fall, the cost difference for WGS and targeted sequencing also decreases. However, despite declining sequencing costs, the analytic costs (monetary and time) will still be larger for WGS experiments in the foreseeable future. Targeted sequencing therefore allows for reduced data generation and analysis costs, or allows for more samples to be sequenced.

Many of the available targeting methods rely on predesigned regions, which may not be suitable to address the scientific questions of a particular experiment. Exome sequencing (ES), for example, is generally limited to protein coding regions of genomes. While investigation of coding sequences can be powerful, significant evidence supports the critical function of the non-coding (and even non-genic) portions of the genome [3,4]. Thus, there is a need for methods that can interrogate other customized subsets of the genome in a variety of organisms.

Hybridization capture technologies [5-8] allow for custom probe designs. The cost of the custom capture probes is generally proportional to the total size of the regions of interest. When regions of interest are large, and focused on a single chromosome, it becomes reasonable to capture the entire chromosome instead of using multiple custom hybridization reactions. Although a single chromosome can be a large target, it is a small fraction of the whole genome. For example, chromosomes in the human genome each make up only 2-8% of the total genome size. Chromosomal flow sorting to isolate specific chromosomes, although technically challenging, has undergone many improvements (for review, see [9,10]). Flow sorting paired with massively-parallel sequencing has been reported in the sequencing of mouse chromosome 17 [11], barley chromosomes 1H [12] and 12 additional arms [13], and wheat chromosomes or arms 1A, 1B, and 1D [14], 5A [15], 7DS [16], 7BS [17], and 4A [18]. This targeting of isolated chromosomes is a powerful approach to understanding complex plant genomes. Flow sorting and sequencing has also been used on human genomes to identify translocation breakpoints in derivative chromosomes [19] and in a method to determine phase across a chromosome [20]. These results suggest FS is a powerful capture method for sequencing single chromosomes.

We present a comparison of Solution Hybrid Selection (SHS) (Agilent SureSelect) and Flow Sorting (FS) capture technologies to target a chromosome of interest for massively-parallel sequencing. We show that FS can be used to target the X chromosome of the human genome for the purpose of identifying genotypes and structural variations. We then compare sequencing efficiency, region of interest coverage, and genotype determination rates of SHS and FS. This comparison will be useful for researchers interested in the targeted sequencing of custom regions of interest, particularly when those regions can be found on a single chromosome.

## Results and discussion

### Targets

**Figure 1:**
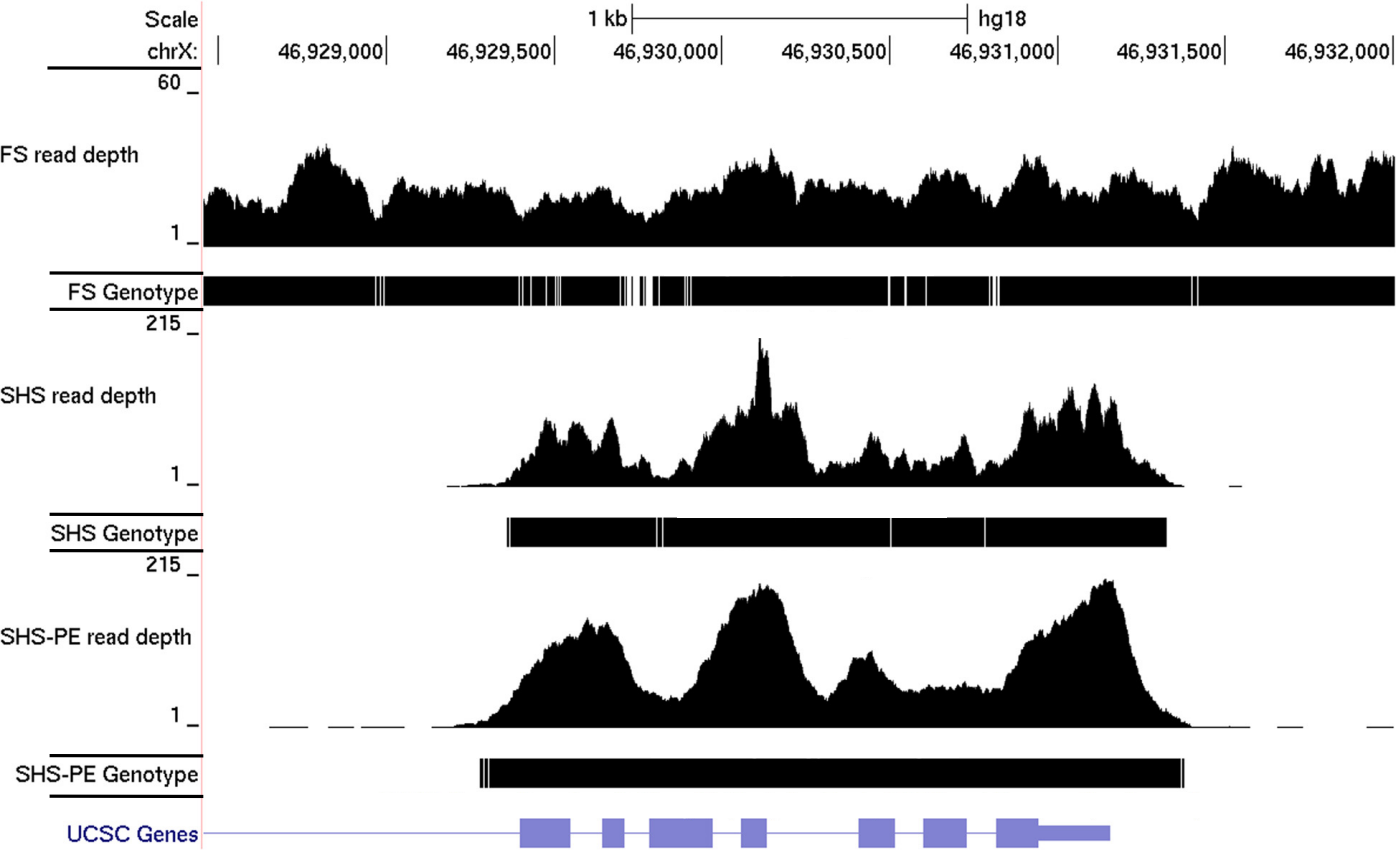
**Illustrative UCSC Genome Browser (**http://genome.ucsc.edu/**) screen capture showing (from top to bottom) chromosome position, read depth from Flow Sort (FS) sequence data, bases covered by high-quality genotype calls in FS sequence data, read depth from Solution Hybrid Selection (SHS) sequence data, bases covered by high-quality genotype calls in SHS sequence data, read depth from Solution Hybrid Selection – Paired End (SHS-PE) sequence data, bases covered by high-quality genotype calls in SHS-PE sequence data, and the UCSC gene models.** Read depth axes show 0–60 reads (FS) and 0–215 reads (SHS).

Human chromosome X from a single individual was targeted by Flow Sorting (FS) or by Solution Hybrid Selection (SHS), and chromosome X from a second individual was targeted with SHS (and sequenced in a paired-end configuration: SHS-PE and SHS-PE low). While FS targeted the entire chromosome X, the SHS probes used here targeted the exons of annotated genes located outside of the pseudo-autosomal regions (PAR). We first determined the overlap of several exon annotation definitions and the regions targeted by SHS. The regions targeted by SHS overlapped 96.3% of the non-PAR Consensus CDS [21] (CCDS) regions, and 81.3% of the non-PAR UCSC known Genes exons (Table 1). Visual inspection of the targeted regions indicated the UTRs were included in the targets (Figure 1). However, the SHS targets were limited to exonic regions and only 2% of chromosome X was targeted (Table 1). To facilitate comparison of SHS and FS we used three region of interest (ROI) definitions: demoX (the regions directly covered by SHS probes), CCDS non-PAR regions, and UCSC non-PAR regions.

**Table 1:** Target metrics

	**Target sizes (bp)**	**Coverage by demoX (%)**
demoX	3,045,718	100
chrX_nonPAR_CCDS	1,129,386	96.3
chrX_nonPAR_UCSC	2,781,987	81.3
chrX	154,913,754	2
chrX_nonPAR	151,874,718	2

Target size in bases, and percentage of target covered by the Solution Hybrid Selection targets (demoX). nonPAR excludes the pseudoautosomal regions (PAR) shared by chromosomes X and Y.

### Flow sorting

**Figure 2:**
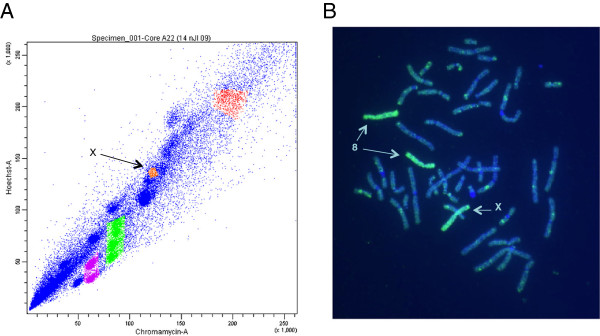
**Bivariate flow sorting and sort verification of chromosome X.** (**A**) Bivariate flow karyotype of human lymphoblastic cell line. The chromosome X peak is indicated with an arrow and highlighted in orange. (**B**) Metaphase chromosomes following hybridization with chromosome X sort material. Note that chromosome X and chromosome 8 were detected. The chromosomes were counterstained with DAPI.

The Hoechst 33258 versus chromomycin bivariate flow karyotype generated from chromosomes isolated from human lymphoblastic cells is shown in Figure 2A. Initial verification of purity of the sorted chromosome X was carried out using degenerate nucleotide primed (DOP)-PCR of the material and painting the probe back onto normal metaphase spreads. The painting probe from the sorted chromosome X peak hybridized to the X chromosome and to chromosome 8, indicating the chromosome X population was not completely resolved from the similarly sized chromosome 8 population (Figure 2B).

### Sequencing results

**Figure 3:**
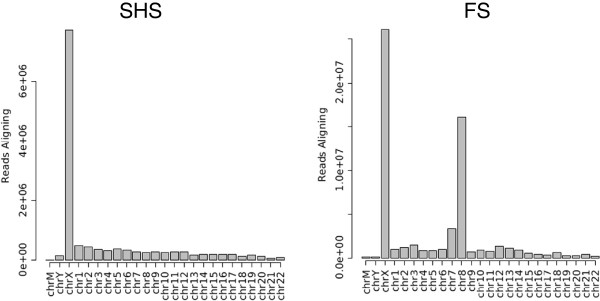
**Number of sequence reads aligning to each chromosome after capture with Solution Hybrid Selection (SHS) or Flow Sort (FS).** Note that FS showed significant off-target alignment to chromosomes 7 (159 Mb) and chromosome 8 (146 Mb) due to size similarity with chromosome X (155 Mb). SHS showed lesser off-target alignment, with amounts corresponding to chromosome size.

Following selection by SHS or FS, libraries were sequenced on an Illumina GAIIx. The SHS capture material was sequenced on a single-end 36 bp lane, which generated a total of 20,896,079 sequence reads (Table 2). Flow sorting targeted the entire chromosome, and more sequence reads were generated to achieve depth of coverage sufficient for genotype determination: three paired-end 76 bp lanes for a total of 92,197,660 reads. A sample from a second individual was subjected to SHS and sequenced on a paired-end 76bp lane. Subsets of read pairs were randomly selected as SHS-PE (to match the total number of reads generated for the initial SHS sample: 20,893,298 reads) or as “SHS-PE low” (to match the total mean base coverage (total sequenced bases/targeted bases) for the FS sample: 1,814,872 reads). Similar percentages of raw reads passed initial chastity filters (76.6% of SHS reads, 90.0% of SHS-PE reads, 80.7% of FS reads). Although the percentage of filtered reads aligning to the genome was similar for each method (80.5% for SHS, 88.6% for SHS-PE, 78.7% for FS), the percentage of filtered reads aligning to the X chromosome was higher for the SHS capture: 46.8% (SHS) and 43.8% (SHS-PE) compared to 33.4% for FS (Table 2). We counted the number of reads aligning to each chromosome, and found that the FS DNA was not only enriched for reads aligning to X, but also for reads aligning to chromosome 8 and, to a lesser extent, chromosome 7 (Figure 3). While SHS reads also aligned to other chromosomes, no chromosomes had higher coverage as observed in the FS data; counts were proportional to the size of the chromosome. Finally, we applied methods to recover non-mapped reads (often containing insertions or deletions) (see Methods), and were able to increase the final number of aligned reads to chromosome X (additional reads added 1.4% of filtered reads for SHS, 1.6% of filtered reads for FS). Total bases sequenced and aligning to chromosome X are listed in Table 2.

**Table 2:** Sequence metrics

	**Total reads**	**Total bases**	**Filtered reads**	**Filtered reads on X**	**Realigned reads on X**	**Realigned bases on X**	**Total mean base coverage**
SHS	20,896,079	752,258,844	15,998,676	7,493,424	7,724,895	278,096,220	247.0x
SHS-PE	20,893,298	1,587,890,648	18,806,032	8,230,111	8,242,645	626,441,020	521.4x
SHS-PE Low	1,814,872	137,937,872	1,633,726	714,915	716,084	54,422,384	45.3x
FS	92,197,660	7,007,022,160	74,367,412	24,835,187	26,040,307	1,979,063,332	45.2x

Read counts for sequence data from Solution Hybrid Selection (SHS), SHS paired end (SHS-PE), SHS paired end low (SHS-PE low) and Flow Sort (FS) capture experiments. Absolute number of read counts and base counts are shown. Total mean base coverage is (total sequenced bases/total targeted bases).

### Sequencing coverage

**Figure 4:**
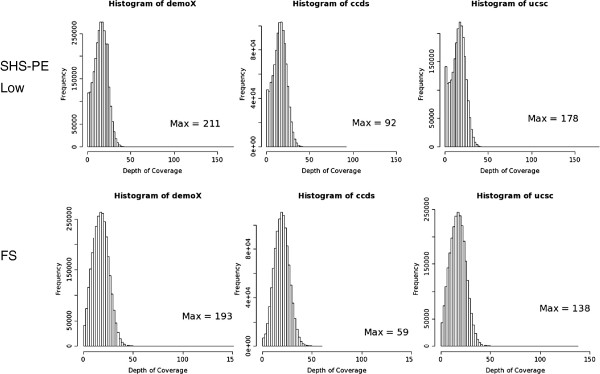
**Distributions of read depths across different regions of interest using Solution Hybrid Selection paired end low (SHS-PE low) or Flow Sort (FS).** The left-tail in the SHS-PE low distributions suggest this method results in more targeted positions with little or no coverage.

Genotype determination requires multiple reads overlapping a single position to give a high probability of observing both alleles at a potential heterozygous site. We therefore calculated the fraction of each ROI covered by ≥10 or ≥20 sequence reads. When the total mean base coverage was similar, (SHS-PE low vs. FS), FS had a higher fraction of bases covered by ≥10 or ≥20 sequence reads (Table 3). To further investigate this, we determined the distributions of depth of coverage at each base across each ROI for FS and SHS-PE low (Figure 4). A broader, skewed distribution of coverage depths was observed for SHS-PE low, due to more bases having the lowest coverage (a left tail in the distribution that was absent in FS). These bases with very low coverage in SHS samples remain even with more reads (Additional file 1: Figure S1), suggesting that the regions are not being effectively captured. This is further illustrated in Figure 1 where the FS read depths, although lower overall, are much more tightly distributed than the SHS read depths.

**Table 3:** Coverage depth

	**≥10x coverage (%)**			**≥20x coverage (%)**			**Genotype coverage (%)**		
	**demoX**	**chrX_nonPAR**	**chrX_nonPAR**	**demoX**	**chrX_nonPAR**	**chrX_nonPAR**	**demoX**	**chrX_nonPAR**	**chrX_nonPAR**
		**CCDS**	**UCSC**		**CCDS**	**UCSC**		**CCDS**	**UCSC**
SHS	87.8	87.9	75.4	83.2	84.4	71.3	86.7	86.8	74.3
SHS-PE	92.0	91.6	81.3	90.2	89.8	78.9	91.6	91.2	80.8
SHS-PE Low	70.7	70.4	61.8	29.0	29.2	27.8	66.4	66.1	58.5
FS	78.2	86.5	77.4	38.9	48.7	37.5	69.9	78.8	68.9

Coverage statistics for sequence data are shown as percentages. “Coverage” displays the percentage of positions in a given target having 10 or more overlapping sequence reads, or 20 or more overlapping sequence reads. “Genotype coverage” displays the percentage of positions in a given target with a high quality genotype determination: a genotype is determined (reference or variant) with a log likelihood score > =10.

### Genotype coverage

Genotypes were determined using a Bayesian algorithm, Most Probable Genotype (MPG) [22]. This algorithm was designed to determine the most probable genotype at a position, and not just whether a variant existed at that position. Therefore, we were able to assess how many positions had sufficient read coverage to reliably detect sequence variants. The samples sequenced were males, which could allow for increased sensitivity as MPG can utilize a single-allele model. However, we determined genotypes using the standard biallelic model (which assumes two chromosomes) in order to allow for broader comparability across any chromosome. We counted the fraction of each ROI covered with high-quality genotype calls (both reference and variant), and found that when total mean base coverage was similar (SHS-PE low and FS), FS had a higher fraction of ROI bases covered (Table 3). This discrepancy was highest in the CCDS target set, and lowest in the demoX ROI set, and was correlated with the > =10x coverage. Increasing sequence amounts in SHS and SHS-PE resulted in higher genotype coverage. We next evaluated the overlap of genotype identifications in SHS and FS across each ROI. Overlap was highest across the non_par CCDS, and lowest across the non_par UCSC (Table 4). Both methods determined the fewest genotypes in the non_par UCSC ROI region.

**Table 4:** Overlap of genotype determinations

	**demoX**	**chrX nonPar CCDS**	**chrX nonPar UCSC**
Covered by both	67.9	75.0	58.2
Unique to FS	2.7	3.8	10.7
Unique to SHS	19.5	11.8	16.1
Missed by both	10.6	9.4	15.0

Percent bases covered by both capture methods, by one method alone, or by neither method.

Overlap is shown for different regions of interest.

### Insertion/deletion variation

**Figure 5:**
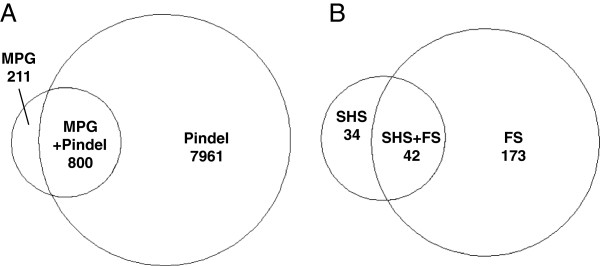
**Overlap of indel determinations.** (**A**) Overlap between indels identified using MPG or Breakdancer/Pindel to analyze FS data. (**B**) Overlap between the same sample using SHS(MPG) and FS(Breakdancer/Pindel), limited to the demoX region + 100 bp.

MPG was also able to determine insertion/deletion (indel) genotypes. We compared the ability to detect indels between the methods by counting variants. As the samples originated from two individuals of northern European descent, the number of events should be similar. More indels (<11 bp) were detected by MPG in the SHS experiments than in the FS experiment. Although different samples were compared, at similar total mean base coverage, SHS-PE low detected 30 indels compared to 28 for FS (Table 5), suggesting ability to detect indels is similar. We also used BreakDancer [23] followed by Pindel [24] to identify indels when paired end data were available. When total mean base coverage was similar, FS had more calls (215) than SHS-PE low (179). However, SHS-PE detected many more indels (818) due to higher sequence coverage. We noted that the BreakDancer/Pindel method resulted in many more indel determinations than MPG. We therefore compared the calls between BreakDancer/Pindel and MPG for each paired end capture experiment, and found that the majority of MPG indel determinations were also made by BreakDancer/Pindel (FS = 79.1%, SHS-PE low = 83.3%, SHS-PE = 93.5%) (Figure 5A, Additional file 1: Figure S2.). However, fewer of the BreakDancer/Pindel determinations were observed with MPG (FS = 9.1%, SHS-PE low = 14.0%, SHS-PE = 33.3%). This suggests that BreakDancer/Pindel was less stringent; indeed, with more sequence data, there were 9.7x more MPG indel calls in SHS-PE compared to SHS-PE low, whereas there were 4.6x more BreakDancer/Pindel calls. The additional data allowed MPG to make more additional indel determinations; many of the BreakDancer/Pindel indel determinations had already been made in the lower coverage data.

**Table 5:** Indel and SV determination

	**Indels - MPG**	**Indels - Pindel**	**Large deletions**	**Large insertions**
SHS	76	-	-	-
SHS-PE	291	818	6	0
SHS-PE	30	179	1	0
LOW				
FS	1,011 (28)	8,761 (215)	38 (2)	3 (0)

Observed variant counts for each class. Pindel calls are divided as follows: Indels: <11bp, large deletions: > = 100bp. The size of large insertions is not determined, as the entire insertion was not covered. FS values in parentheses indicate variants detected in the demoX region + 100bp flanking.

We also compared the overlap of indel determination in SHS and FS in the same sample. Although SHS used shorter, unpaired sequence reads, more MPG indels were determined than in FS (due to higher total mean base coverage for SHS). We therefore compared the FS BreakDancer/Pindel calls to SHS MPG calls (Figure 5B) and observed that, as above within a sample, the majority of SHS MPG indel determinations were also observed by FS BreakDancer/Pindel. This suggests both SHS and FS are sensitive to smaller indels, particularly with increasing sequence read depth and length.

### Structural variation

**Figure 6:**
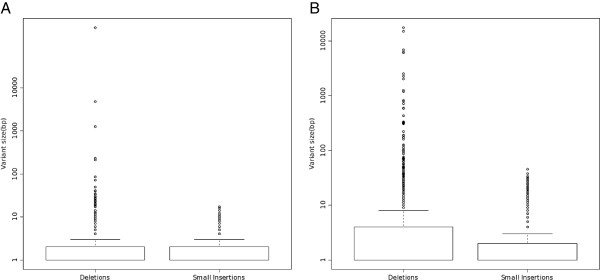
**Evaluation of structural variant discovery.** Boxplots showing size of deletions and small insertions identified using SHS-PE (**A**) and FS (**B**) sequence data. In all cases, the median variant size is 1 (the lowest line). The top of the box is the 75th percentile, and the whisker is the highest value still less than the 75th percentile + (1.5 * interquartile range). Note the y-axis uses a log scale. A 25 Mb (SHS-PE) and a 27Mb (FS) deletion were omitted, as they were far outliers that appeared to be false positive determinations.

The ability to detect structural variation (SV) is a distinct advantage of whole genome sequencing. Flow sorted chromosomes are subjected to random shotgun sequencing, resulting in even coverage across the chromosome (example in Figure 1) similar to whole genome sequencing. The FS experiment resulted in 12.4-fold average physical coverage, suggesting that structural variants could be identified. To compare structural variation in FS and SHS-PE, we combined two approaches to reduce false positive calls. We first identified regions with atypical insert sizes using BreakDancer [23]. These candidate regions were then supplied to Pindel [24], which detected structural variants based on different parts of a single sequence read aligning to different locations in the genome. The vast majority of detected deletions and insertions were small (Figure 6), and were discussed above. Deletion sizes covered a larger range (FS = 1–17,263 bp, SHS-PE = 1–245,716 bp) than did small insertions (FS = 1–46 bp, SHS-PE = 1–17 bp). However, the exact size of larger insertions cannot be determined when the inserted sequence is longer than a sequence read.

Although FS identified many more SVs overall (Table 5), only two large deletions overlapped with the demoX target, compared to one large deletion for SHS-PE low. With more sequence, SHS-PE identified six large deletions, but no large insertions. To better understand specificity, we compared different size classes of insertion and deletion to the Database of Genomic Variants (DGV, http://projects.tcag.ca/variation/, [25]) as in [24]. Medium deletions (100 bp-1 kb) detected by SHS-PE (2) and SHS-PE low (1) were not observed in DGV, but 22/26 (84.6%) observed in FS overlapped a DGV entry. Large deletions (> = 1 kb) were observed in FS and SHS-PE, and half were observed in DGV (FS = 6/12, SHS-PE = 2/4). The overlap of the FS deletions with DGV exceeded that observed in [24] (67.8% for medium deletions, 43.4% for large) suggesting FS is able to reliably detect SVs (SV detection by SHS was limited by the target size, so the specificity of SV detections from this method is not clear.)

## Conclusions

When evaluating targeted sequencing methods, it is important to consider the genomic regions of interest. We have examined both FS, which targets an entire chromosome, and SHS, which targets defined regions. Our comparisons focused on evaluating capture method effectiveness. Of the filtered reads generated, FS had a lower percentage aligned to chromosome X. This was due to the large off-target FS capture of chromosomes 7 and 8, which is caused by imperfect separation of chromosomes when performing flow sorting (Figure 2). The degree of off-target capture depends on the chromosome being investigated, as some chromosomes can be separated more effectively than others. For example, chromosomes 1, 2, 3, and 4 are typically resolved as single peaks whereas chromosomes 9, 10, 11, and 12 are clustered and less easily separated from each other. Recently, the use of increased power settings for the laser in the cell sorter was shown to improve the resolution of the flow karyotypes (even for chromosomes 9–12) and is therefore a more attractive approach for projects involving massively parallel sequencing of flow sorted chromosomes [26]. SHS results in more on-target sequence reads than FS, but it too results in significant amounts of off-target sequence. Sequencing efficiency (the amount of sequence data required to achieve a given coverage across all bases) also contributes to the effectiveness of capture. We evaluated efficiency by examining the distribution of read depths across ROIs. The SHS method was less efficient, as a broader distribution of read depths was observed. In contrast, FS had a tighter distribution of coverage. More importantly, when total mean base coverage was equivalent, FS had a slightly higher genotype determination rate. Although adding more sequence to an SHS experiment increased the genotype determination rate, there were still a number of bases with little or no sequence coverage, likely due to poor capture. Genotypes could be determined at a majority of bases by both methods, but many bases (up to 19.5%) were covered by one method alone, suggesting a combined approach could be used to increase sensitivity. Both methods were amenable to indel and larger SV determination, and similar numbers were observed within the region of interest. While FS targeting may be less efficient than SHS, and SHS sequence efficiency may be less than FS, the two methods are effective for determining genotypes (with FS being slightly more sensitive.) SHS has a design advantage in being able to target regions smaller than a single chromosome. It is therefore important to consider both capture method effectiveness as well as the target design when planning a targeted sequencing experiment.

Experimental cost and ease of use are also important when choosing a sequencing method. In this case, the cost of custom SHS probes for a ~3 Mb target region is similar to that of whole exome SHS probes. In order to cover a whole chromosome, multiple larger probe designs would be required. For example, while list prices (at the time of writing) for hybridization capture reagents range from $450-$1250 per sample for 3 Mb, these prices rise to $4,500-$7,000 per sample to cover chromosome X (150 Mb). Both methods require standard sequencer-specific library preparation. Standard library preparations allow for indexing, which can be used to combine multiple samples for sequencing in order to take advantage of newer high-output sequencing instruments. If we assume the need for 100x total mean base coverage for sensitive genotype determination, this would require at least 155 million 100 base pair reads for chromosome X. As of this writing, a current, widely used sequencer (Illumina HiSeq2000) can generate up to 375 million paired-end reads per lane, making the ability to pool samples essential. The SHS capture method was straightforward, and although some steps required long incubations, hands-on time was relatively low. The FS experiments required access to a flow sorting instrument, as well as the technical expertise to properly perform the chromosomal separations. In addition to this cost, sorting experiments are time consuming and require a large number of mitotic cells, which may be a barrier to high-throughput use of this method.

Although both methods are capable of selecting regions of interest for massively-parallel sequencing, one may certainly be more appropriate than the other depending on the experimental goal. If investigators are targeting sub-chromosomal regions, SHS reagents and sequencing will be less costly, and easier to perform. However, if an investigator wants to sequence larger regions of interest on the same chromosome, or wishes to sequence structurally abnormal “marker” chromosomes, FS may be more appealing. The higher sequence efficiency of FS may partially offset the need to sequence a greater amount of captured DNA. Finally, custom SHS kits include reagents for a minimum sample batch size, and FS may offer a cost advantage when only one or two samples are needed. Conversely, SHS is more suitable for larger sample numbers as it is tailored for high-throughput experiments.

The ever-decreasing costs of massively-parallel sequencing are making whole genome sequencing more practical. However, there are still many advantages to targeting smaller subsets of the genome. Experimental cost is, as of this writing, still lower for targeted sequencing, even for a complete chromosome. The amount of data requiring analysis and storage is much lower for targeted sequencing experiments. Therefore, for a given financial and computational budget, more samples can be analyzed with targeting, increasing the power of an experiment. The lower analytical burden can also result in faster return of results. We have shown that SHS and FS are both effective at focusing sequencing efforts on a targeted subset of the genome. Each method fits specific needs, which will allow researchers with a wide variety of experimental designs and resources to take advantage of this powerful new technology.

## Methods

### Sample

The subjects were part of a National Institutes of Health IRB approved study (#94-HG-0193), and provided informed consent. A lymphoblastic cell line was cultured to achieve the high number of cells needed for a flow sorting experiment. Cells were grown and chromosomes were prepared as described [27].

### Flow sorting and in-situ hybridization

Chromosome preparations were sorted as described [27]. Approximately 1.1 × 10^6^ chromosomes were sorted using a dual laser cell sorter (FACS DiVa, Becton-Dickinson). This system allowed a bivariate analysis of both DNA content and base-pair composition.

For sort verification, approximately 500 chromosomes were sorted directly into PCR tubes containing 30 μL of water. The 6MW primer [28] was used in a primary degenerate oligonucleotide primed PCR (DOP-PCR) to amplify the DNA and then in a secondary PCR reaction to label the chromosomal DNA with biotin-dUTP. *In situ* hybridization and probe detection was carried out following common fluorescence *in situ* hybridization (FISH) procedures. Briefly, 300–400 ng of biotinylated PCR product was precipitated with 10 μg of human COT-1 (Invitrogen, Grand Island, NY)) and then dissolved in 14 μl hybridization buffer. Following hybridization, slides were washed and the biotinylated probe was detected with avidin coupled with fluorescein (Vector Laboratories, Burlingame, CA).

### Post-sorting DNA purification

DNA was prepared from 250 μL of flow sorted material by adding 15 μL 0.25M EDTA/10% sodium lauroyl sarcosine and 2.5 μL proteinase K (20ng/ml), and incubating overnight at 42°C. Following overnight incubation, 0.17 mM phenylmethylsulfonyl fluoride (PMSF) was added and incubated for 40 minutes at room temperature. Next, the DNA was purified through QIAamp DNA Micro Kit (Qiagen, Valencia, CA) following the manufacturer’s recommended protocol. Purified DNA was used as template for shearing on the Covaris adaptive focused acoustics (AFA) sonicator (Covaris, Inc., Woburn, MA).

### Solution hybrid selection

The SHS technique was performed using the SureSelect Human X Chromosome demonstration kit (Agilent Technologies Inc., Santa Clara, CA) according to the manufacturer’s instructions, with modifications as in [22].

### Sequencing

Libraries were prepared and sequenced on a Genome Analyzer IIx (Illumina Inc., San Diego, CA) according to the manufacturer’s protocols.

### Data analyses

Initial analysis was carried out using the standard Illumina software, including alignment of sequence reads with ELAND.

A secondary analysis was performed to recover reads that may not have mapped well due to insertions or deletions. For the paired-end data, reads were placed in bins of approximately 100 kilobases along the genome. If one member of a read pair was unaligned, it was placed in the same bin as its mapped mate. Reads were then realigned to the subsection of the genome using a gap-aware alignment program, cross_match (http://www.phrap.org/phredphrapconsed.html). Single-end sequencing was performed for the SHS capture, and so the above approach was not effective (there were no mate pairs to rescue unaligned reads.) We therefore used cross_match to realign all of the unmapped reads against the entire human genome, and then combined the realigned cross_match reads with the reads aligned by ELAND.

In both cases, cross_match and ELAND outputs were converted to the SAM/BAM format [29], and genotypes were determined using the Most Probable Genotype (MPG) algorithm [22]. Structural variants were detected from paired-end data by first running BreakDancer [23] on the realigned BAM file (described above) using default settings. This output was then used to guide variant detection using Pindel [24] (Illumina-PairEnd mode, median insert size of 146 for FS, 182 for SHS-PE and SHS-PE low). Data analysis and comparison was performed using custom Perl scripts, as well as BED file manipulation programs from the bx-python package (https://bitbucket.org/james_taylor/bx-python/wiki/Home) and bedTools [30], and VCF file manipulation programs from VCFtools (vcftools.sourceforge.net). Overlap of SVs with DGV was counted when a 50% reciprocal overlap was observed. Area-proportional Venn diagrams were prepared using the web tool 3Venn (https://www.cs.kent.ac.uk/people/staff/pjr/EulerVennCircles/EulerVennApplet.html).

## Abbreviations

SHS: Solution hybrid selection; FS: Flow sorting; WGS: Whole genome sequencing; ROI: Regions of interest; Mb: Megabases; PAR: Pseudo-autosomal regions; MPG: Most probable genotype.

## Competing interests

LGB is a paid editor for the American Journal of Medical Genetics and is an uncompensated advisor to the Illumina Corporation as part of his official U.S. Government duties.

## Authors’ contributions

JKT performed the data analysis, prepared the manuscript, and assisted with the SHS capture. JJJ performed the SHS capture and assisted with the manuscript preparation. SA, MP, and GS performed the flow sorting and prepared the Illumina library for the sorted chromosome X. Sequencing was performed at NISC. PM, JCM, and LGB helped design the experiments and revised the manuscript. All authors read and approved the final manuscript.

## Supplementary Material

Additional file 1: Figure S1 Distributions of read depths across different regions of interest using Solution Hybrid Selection (SHS) or Flow Sort (FS). Although FS showed lower average coverage, the coverage distribution was much sharper. **Figure S2.** Overlap of MPG and Breakdancer/Pindel calls in the SHS-PE (A) and SHS-PE low (B) libraries. Click here for file

## References

[B1] Teer JK Mullikin JC Exome sequencing: the sweet spot before whole genomes Hum Mol Genet 2010 19 R2 R145 151 10.1093/hmg/ddq333 20705737 PMC2953745

[B2] Biesecker LG Shianna KV Mullikin JC Exome sequencing: the expert view Genome Biol 2011 12 9 128 10.1186/gb-2011-12-9-128 21920051 PMC3308041

[B3] Birney E Stamatoyannopoulos JA Dutta A Guigo R Gingeras TR Margulies EH Weng Z Snyder M Dermitzakis ET Thurman RE Identification and analysis of functional elements in 1% of the human genome by the ENCODE pilot project Nature 2007 447 7146 799 816 10.1038/nature05874 17571346 PMC2212820

[B4] Dunham I Kundaje A Aldred SF Collins PJ Davis CA Doyle F Epstein CB Frietze S Harrow J Kaul R An integrated encyclopedia of DNA elements in the human genome Nature 2012 489 7414 57 74 10.1038/nature11247 22955616 PMC3439153

[B5] Albert TJ Molla MN Muzny DM Nazareth L Wheeler D Song X Richmond TA Middle CM Rodesch MJ Packard CJ Direct selection of human genomic loci by microarray hybridization Nat Methods 2007 4 11 903 905 10.1038/nmeth1111 17934467

[B6] Okou DT Steinberg KM Middle C Cutler DJ Albert TJ Zwick ME Microarray-based genomic selection for high-throughput resequencing Nat Methods 2007 4 11 907 909 10.1038/nmeth1109 17934469

[B7] Gnirke A Melnikov A Maguire J Rogov P LeProust EM Brockman W Fennell T Giannoukos G Fisher S Russ C Solution hybrid selection with ultra-long oligonucleotides for massively parallel targeted sequencing Nat Biotechnol 2009 27 2 182 189 10.1038/nbt.1523 19182786 PMC2663421

[B8] Bainbridge MN Wang M Burgess DL Kovar C Rodesch MJ D'Ascenzo M Kitzman J Wu YQ Newsham I Richmond TA Whole exome capture in solution with 3Gbp of data Genome Biol 2010 11 6 R62 10.1186/gb-2010-11-6-r62 20565776 PMC2911110

[B9] Ibrahim SF van den Engh G High-speed chromosome sorting Chromosome Res 2004 12 1 5 14 14984097 10.1023/b:chro.0000009328.96958.a6

[B10] Dolezel J Vrana J Safar J Bartos J Kubalakova M Simkova H Chromosomes in the flow to simplify genome analysis Funct Integr Genomics 2012 12 3 397 416 10.1007/s10142-012-0293-0 22895700 PMC3431466

[B11] Sudbery I Stalker J Simpson JT Keane T Rust AG Hurles ME Walter K Lynch D Teboul L Brown SD Deep short-read sequencing of chromosome 17 from the mouse strains A/J and CAST/Ei identifies significant germline variation and candidate genes that regulate liver triglyceride levels Genome Biol 2009 10 10 R112 10.1186/gb-2009-10-10-r112 19825173 PMC2784327

[B12] Mayer KF Taudien S Martis M Simkova H Suchankova P Gundlach H Wicker T Petzold A Felder M Steuernagel B Gene content and virtual gene order of barley chromosome 1H Plant Physiol 2009 151 2 496 505 10.1104/pp.109.142612 19692534 PMC2754631

[B13] Mayer KF Martis M Hedley PE Simkova H Liu H Morris JA Steuernagel B Taudien S Roessner S Gundlach H Unlocking the barley genome by chromosomal and comparative genomics Plant Cell 2011 23 4 1249 1263 10.1105/tpc.110.082537 21467582 PMC3101540

[B14] Wicker T Mayer KF Gundlach H Martis M Steuernagel B Scholz U Simkova H Kubalakova M Choulet F Taudien S Frequent gene movement and pseudogene evolution is common to the large and complex genomes of wheat, barley, and their relatives Plant Cell 2011 23 5 1706 1718 10.1105/tpc.111.086629 21622801 PMC3123954

[B15] Vitulo N Albiero A Forcato C Campagna D Dal Pero F Bagnaresi P Colaiacovo M Faccioli P Lamontanara A Simkova H First survey of the wheat chromosome 5A composition through a next generation sequencing approach PLoS One 2011 6 10 e26421 10.1371/journal.pone.0026421 22028874 PMC3196578

[B16] Berkman PJ Skarshewski A Lorenc MT Lai K Duran C Ling EY Stiller J Smits L Imelfort M Manoli S Sequencing and assembly of low copy and genic regions of isolated Triticum aestivum chromosome arm 7DS Plant Biotechnol J 2011 9 7 768 775 10.1111/j.1467-7652.2010.00587.x 21356002

[B17] Berkman PJ Skarshewski A Manoli S Lorenc MT Stiller J Smits L Lai K Campbell E Kubalakova M Simkova H Sequencing wheat chromosome arm 7BS delimits the 7BS/4AL translocation and reveals homoeologous gene conservation Theor Appl Genet 2012 124 3 423 432 10.1007/s00122-011-1717-2 22001910

[B18] Hernandez P Martis M Dorado G Pfeifer M Galvez S Schaaf S Jouve N Simkova H Valarik M Dolezel J Next-generation sequencing and syntenic integration of flow-sorted arms of wheat chromosome 4A exposes the chromosome structure and gene content Plant J 2012 69 3 377 386 10.1111/j.1365-313X.2011.04808.x 21974774

[B19] Chen W Kalscheuer V Tzschach A Menzel C Ullmann R Schulz MH Erdogan F Li N Kijas Z Arkesteijn G Mapping translocation breakpoints by next-generation sequencing Genome Res 2008 18 7 1143 1149 10.1101/gr.076166.108 18326688 PMC2493403

[B20] Yang H Chen X Wong WH Completely phased genome sequencing through chromosome sorting Proc Natl Acad Sci USA 2011 108 1 12 17 10.1073/pnas.1016725108 21169219 PMC3017199

[B21] Pruitt KD Harrow J Harte RA Wallin C Diekhans M Maglott DR Searle S Farrell CM Loveland JE Ruef BJ The consensus coding sequence (CCDS) project: Identifying a common protein-coding gene set for the human and mouse genomes Genome Res 2009 19 7 1316 1323 10.1101/gr.080531.108 19498102 PMC2704439

[B22] Teer JK Bonnycastle LL Chines PS Hansen NF Aoyama N Swift AJ Abaan HO Albert TJ Margulies EH Green ED Systematic comparison of three genomic enrichment methods for massively parallel DNA sequencing Genome Res 2010 20 10 1420 1431 10.1101/gr.106716.110 20810667 PMC2945191

[B23] Chen K Wallis JW McLellan MD Larson DE Kalicki JM Pohl CS McGrath SD Wendl MC Zhang Q Locke DP BreakDancer: an algorithm for high-resolution mapping of genomic structural variation Nat Methods 2009 6 9 677 681 10.1038/nmeth.1363 19668202 PMC3661775

[B24] Ye K Schulz MH Long Q Apweiler R Ning Z Pindel: a pattern growth approach to detect break points of large deletions and medium sized insertions from paired-end short reads Bioinformatics 2009 25 21 2865 2871 10.1093/bioinformatics/btp394 19561018 PMC2781750

[B25] Iafrate AJ Feuk L Rivera MN Listewnik ML Donahoe PK Qi Y Scherer SW Lee C Detection of large-scale variation in the human genome Nat Genet 2004 36 9 949 951 10.1038/ng1416 15286789

[B26] Ng BL Carter NP Laser excitation power and the flow cytometric resolution of complex karyotypes Cytometry A 2010 77 6 585 588 20506467 10.1002/cyto.a.20904 PMC4131141

[B27] Stanyon R Stone G Phylogenomic analysis by chromosome sorting and painting Methods Mol Biol 2008 422 13 29 10.1007/978-1-59745-581-7_2 18629658

[B28] Telenius H Pelmear AH Tunnacliffe A Carter NP Behmel A Ferguson-Smith MA Nordenskjold M Pfragner R Ponder BA Cytogenetic analysis by chromosome painting using DOP-PCR amplified flow-sorted chromosomes Genes Chromosom Cancer 1992 4 3 257 263 10.1002/gcc.2870040311 1382568

[B29] Li H Handsaker B Wysoker A Fennell T Ruan J Homer N Marth G Abecasis G Durbin R The Sequence Alignment/Map format and SAMtools Bioinformatics 2009 25 16 2078 2079 10.1093/bioinformatics/btp352 19505943 PMC2723002

[B30] Quinlan AR Hall IM BEDTools: a flexible suite of utilities for comparing genomic features Bioinformatics 2010 26 6 841 842 10.1093/bioinformatics/btq033 20110278 PMC2832824

