# Supplementary material for: Massively-parallel sequencing of genes on a single chromosome: a comparison of solution hybrid selection and flow sorting
Source: BMC Genomics. 2013 Apr 15;14:253. doi: 10.1186/1471-2164-14-253 (PMC3637801; doi:10.1186/1471-2164-14-253)
Supplement: Additional file 1: Figure S1 — Distributions of read depths across different regions of interest using Solution Hybrid Selection (SHS) or Flow Sort (FS). Although FS showed lower average coverage, the coverage distribution was much sharper. Figure S2. Overlap of MPG and Breakdancer/Pindel calls in the SHS-PE (A) and SHS-PE low (B) libraries. [file 1471-2164-14-253-S1.pdf]

Supp Fig 1.
Distributions of read depths across different regions of interest using Solution Hybrid Selection (SHS) or Flow Sort (FS). Although FS showed lower average coverage, the coverage distribution was much sharper.

Supp. Fig 2.
Overlap of MPG and Breakdancer/Pindel calls in the SHS-PE (A) and SHS-PE low (B) libraries.

Supp. Fig. 1

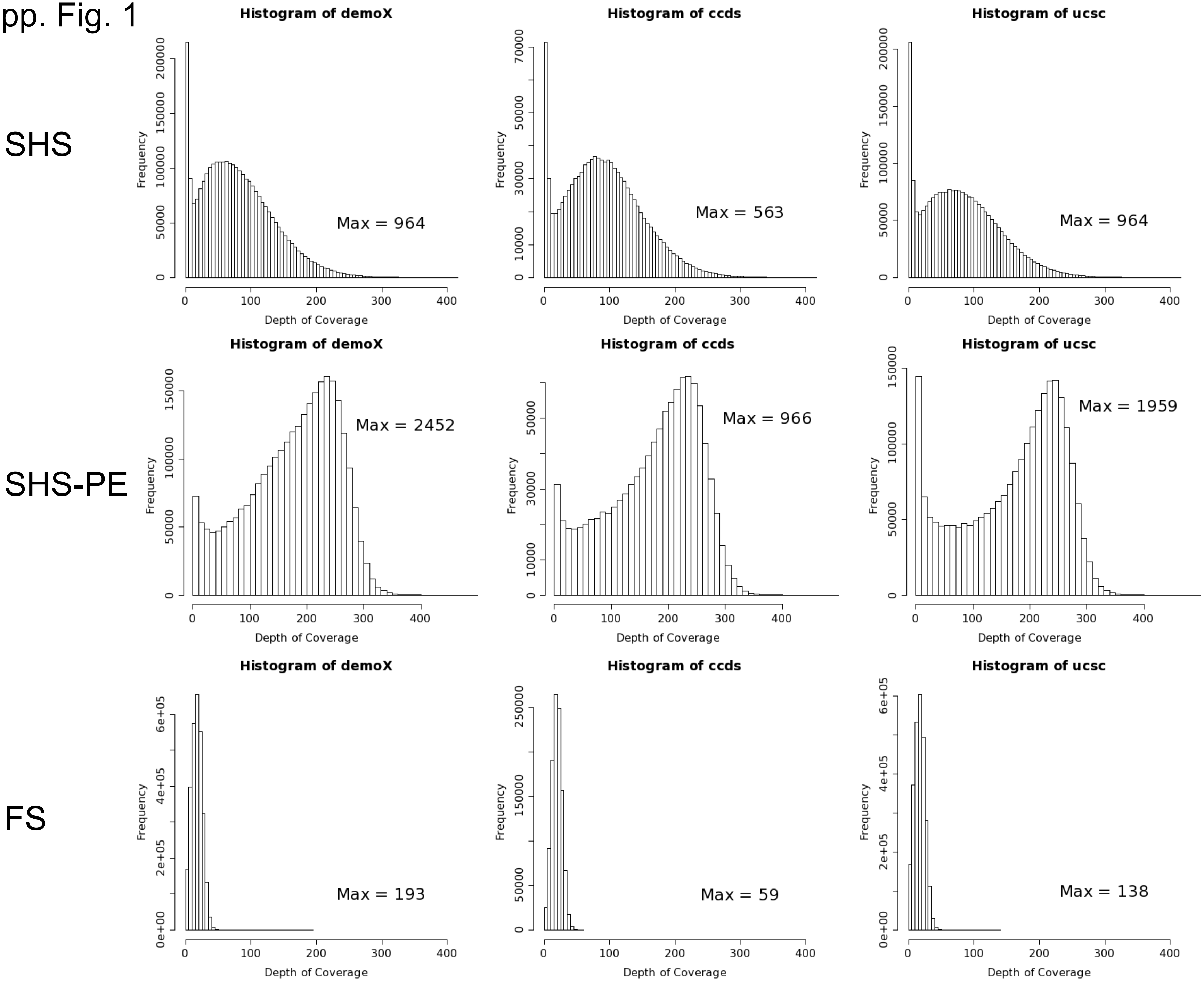
SHS
SHS-PE
FS
Histogram of demoX
Histogram of ccds
Histogram of ucsc
Frequency
Depth of Coverage
Max = 964
Max = 563
Max = 964
Max = 2452
Max = 966
Max = 1959
Max = 193
Max = 59
Max = 138

Supp. Fig. 2

A.

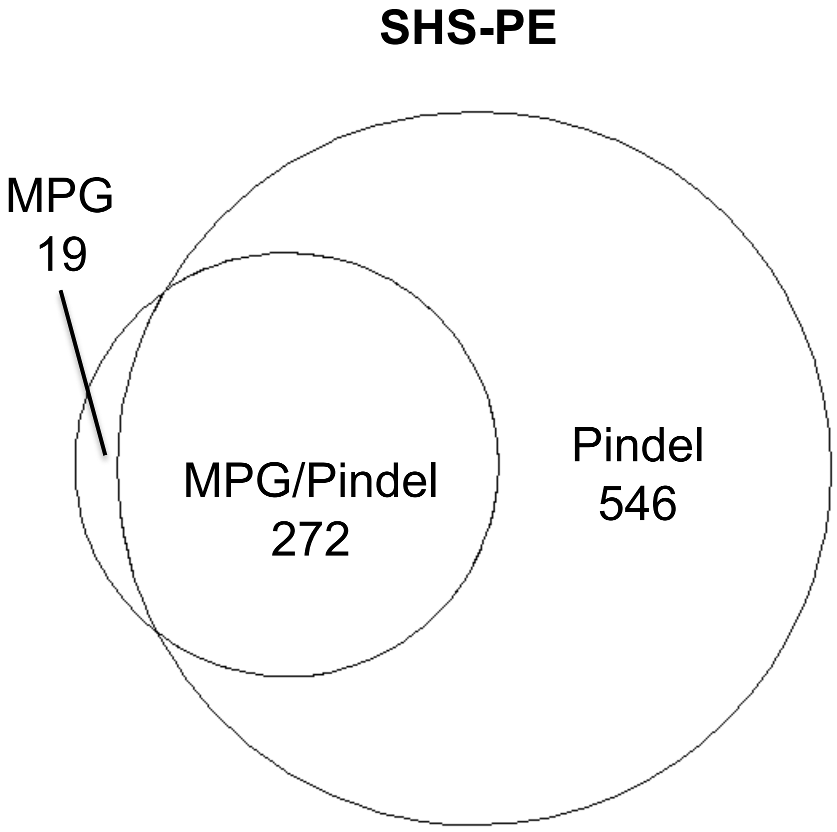
SHS-PE
MPG
19
MPG/Pindel
272
Pindel
546

B.

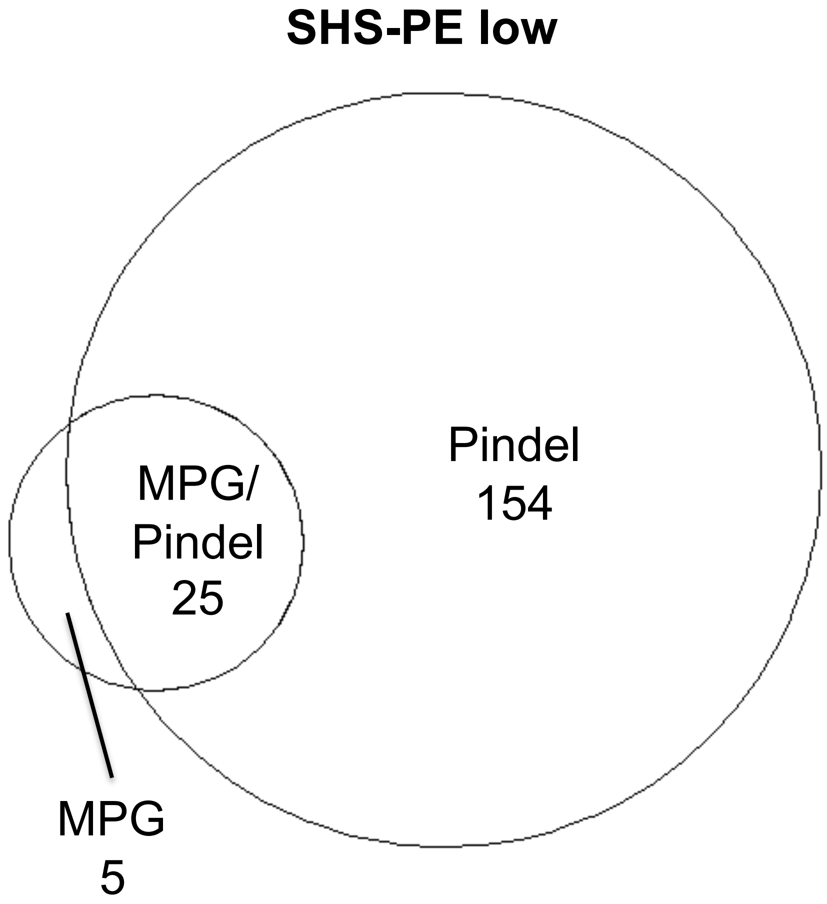
SHS-PE low
MPG/
Pindel
25
Pindel
154
MPG
5
